# *QuickStats:* Percentage* of Youths Aged 2–19 Years Consuming Any Fast Food^†^ on a Given Day, by Race and Hispanic Origin^§^ — National Health and Nutrition Examination Survey, 2013–2016

**DOI:** 10.15585/mmwr.mm6740a8

**Published:** 2018-10-12

**Figure:**
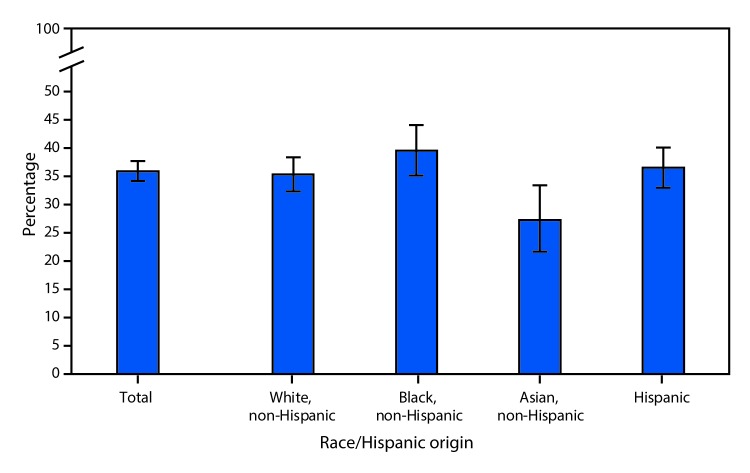
During 2013–2016, 36.0% of youths aged 2–19 consumed fast food on a given day. Non-Hispanic Asian youths (27.3%) had a lower percentage of fast food consumption on a given day, compared with non-Hispanic black (39.6%), Hispanic (36.6%), and non-Hispanic white (35.4%) youths. There were no significant differences in fast food consumption on a given day among non-Hispanic white, non-Hispanic black, and Hispanic youths.

**Sources: **National Center for Health Statistics Data Brief No. 322. https://www.cdc.gov/nchs/products/databriefs/db322.htm; National Center for Health Statistics, National Health and Nutrition Examination Survey Data, 2013–2016. https://www.cdc.gov/nchs/nhanes.htm.

